# New Transcriptional Reporters to Quantify and Monitor PPAR*γ* Activity

**DOI:** 10.1155/2017/6139107

**Published:** 2017-11-01

**Authors:** Séverine A. Degrelle, Hussein Shoaito, Thierry Fournier

**Affiliations:** ^1^INSERM, UMR-S1139, Faculté de Pharmacie de Paris, 75006 Paris, France; ^2^Université Paris Descartes, Sorbonne Paris Cité, 75006 Paris, France; ^3^Fondation PremUp, 75006 Paris, France

## Abstract

The peroxisome-proliferator-activated-receptor-*γ* (PPAR*γ*) is a member of the nuclear receptor superfamily that plays a critical role in diverse biological processes, including adipogenesis, lipid metabolism, and placental development. To study the activity of PPAR*γ*, we constructed two new reporter genes: a fluorescent GFP-tagged histone-2B (PPRE-H2B-eGFP) and a secreted nanoluciferase (PPRE-pNL1.3[secNluc]). This study demonstrates their usage to monitor PPAR*γ* activity in different cell types and screen for PPAR*γ*'s potential ligands.

## 1. Introduction

PPAR*γ* is an isoform of the PPAR subset of nuclear receptors that also includes PPAR*α* and PPAR*β*. They all bind to DNA as heterodimers with retinoid X receptors (RXR). The heterodimers activate the expression of their target genes by binding to peroxisome proliferator response elements (PPREs), which are composed of direct tandem repeats of a consensus sequence spaced by a single nucleotide. Such PPREs are found in genes that are involved in lipid metabolism and homeostasis [1]. PPAR*γ* is a ligand-activated transcription factor that is involved in embryonic development [2, 3], lipid metabolism [4, 5], insulin resistance [6], inflammation [6], immune response, and differentiation of several tissues, including placenta and trophoblast differentiations [2, 7]. The ligand-binding domain (LBD) of PPAR*γ* has a larger and more accessible tertiary structure than that of many other nuclear receptors, enabling the binding of a wide spectrum of ligands [8].

Within the last two decades, PPAR*γ* has become a focus of attention as a transcription factor implicated in metabolic syndrome [9]. Indeed, PPAR*γ* has important roles in pathologies, such as obesity [10], cardiovascular diseases [11, 12], type 2 diabetes [10], atherosclerosis [13], or lipodystrophy [14] because it has lipophilic compounds as ligands: fatty acids and their derivatives, for instance. Thiazolidinediones (TZDs) are synthetic ligands (potent activators of PPAR*γ*) that have been used in treating type 2 diabetes but were finally withdrawn due to critical cardiovascular diseases and bladder cancers as side effects [15]. It is thus of general interest to identify new PPAR*γ*-modulators with less or no side effects.

PPARs have a relatively high basal activity [16]. To date, two approaches have been described to investigate the activity of PPAR*γ*, based on (i) cell transfection with a nonsecreted luciferase reporter gene (transiently in cells of interest [17] or stably using cell lines [18]) and (ii) a humanized in vivo reporter model (*Xenopus laevis* [19]).

Historically, the luciferase (Luc) gene has been successfully used for many years (PPREx3-TK-luc, PPRE-Luc, e.g., [20, 21]), but to measure Luc expression the cells need to be lysed so that a different well is needed at each time point of the assay.

More recently, the green fluorescent protein (GFP or eGFP) has become the reporter of choice for in vitro time lapse imaging and in vivo analysis [19]. This inert reporter is excited by UV light (395 nm) and emits in green light (509 nm) making it easier to visualize using fluorescence microscopy.

Given the importance of PPAR*γ* as a key regulator in several tissues, it is essential to develop a new method to quantify and monitor the activity of PPAR*γ* in any cell types. While PPAR*γ* transcriptional nonsecreted luciferase reporters do exist [20, 21], nuclear-localized fluorescent and secreted luciferase reporters suitable for live imaging (and cell sorting) or quantification without cell lysis are lacking. These tools will present an advantage especially when the number of cells is limited (e.g., primary cultures), or to perform a time course. Furthermore, at the end of the assay, cells can be used for additional experiments (RT-qPCR, western blot).

The current work describes the construction and evaluation of two novel reporters, one based on GFP (PPRE-H2B-eGFP) and the other on a secreted luciferase (PPRE-pNL1.3[secNluc]). To quantify the transcriptional responses elicited from these reporters, we used the well-characterized agonist (GW1929) and antagonist (GW9662) of PPAR*γ* in human trophoblasts, where PPAR*γ* is activated during their differentiation [22]. The exchange and endocrine tissue of the human placenta is the syncytiotrophoblast (ST). It is renewed all along pregnancy by fusion with the underlying villous cytotrophoblast (VCT). This fusion process can be studied in vitro, using human primary cultures of VCT. The functionality of the ST is assessed by the measurement of hCG secretion in the supernatant [23].

Our results verified the benefits of these 2 reporters in evaluating different PPAR*γ* ligands taking into account different cell specificities.

## 2. Materials and Methods

### 2.1. Ethical Statement

The study was performed according to the Declaration of Helsinki. Placentas were obtained with the patients' written informed consent. The protocol was approved by the local ethics committee (CPP 2015-mai-13909). Placental tissues were obtained from women with uncomplicated pregnancy undergoing normal Cesarean section at the Cochin Port-Royal, Antony and Montsouris maternity units (Paris, France).

### 2.2. Cell Culture

Villous cytotrophoblasts (VCT) were isolated from human term placentas as previously described [24–26]. Briefly, several cubic millimeters of the basal plate surface were removed sharply. Villous tissue was gently scraped free from vessels and connective tissue using forceps. After washing thoroughly two times with DMEM and once in 1x Ca2+, Mg2+-free HBSS, the tissue was cut into small pieces. About 15 grams of minced tissues was digested one time in 50 ml of a filtered digestion enzyme medium (containing 500 mg of trypsin powder (Difco), 17.5 ml of fat-free milk, 250 *μ*l DNase I (50 U/ml), 250 *μ*l 0.1 M MgCl2, and 250 *μ*l 0.1 M CaCl2 in 250 ml warm 1x Ca2+, Mg2+-free HBSS) at 37°C for 30 min for a first cycle in a shaking incubator (70 rpm). Then the tissues were incubated with 30 ml of the digestion medium for 10 mins for up to 5 cycles in same conditions. Enzymatic degradation was monitored under light microscopy and stopped by filtering into 50 ml tubes containing 5% FBS. The mix was filtered through a 40 *μ*m strainer (Thermo Fisher Scientific) to remove contaminating tissue debris. Cell suspensions were collected and centrifuged at 1200 rpm for 10 min at room temperature. Cell pellets were suspended in 3 ml of DMEM, layered on the top of a preformed Percoll gradient (60%, 50%, 45%, 35%, 30%, 20%, and 10%) and centrifuged at 2500 rpm at room temperature without braking for 20 minutes. The layer between the 45% and 35% of Percoll containing trophoblast cells was collected, suspended in DMEM, and centrifuged at 1200 rpm at room temperature for 10 min. The resulting cell pellet was suspended in complete DMEM (1% glutamine, 1% PS, and 10% FCS) and the cells were counted using a TC20™ Automated Cell Counter (Biorad). The cells were seeded in triplets onto either a sterile 24-well plate (250.000/well, Corning) for nanoluciferase luciferase assay (PPRE-pNL1.3 transfection) or sterile 12-well cell culture-treated plastic slides (40.000/well, Ibidi) for fluorescent experiments (immunofluorescence and PPRE-H2B-eGFP transfection).

### 2.3. Plasmid Constructs

**Figure 1:**
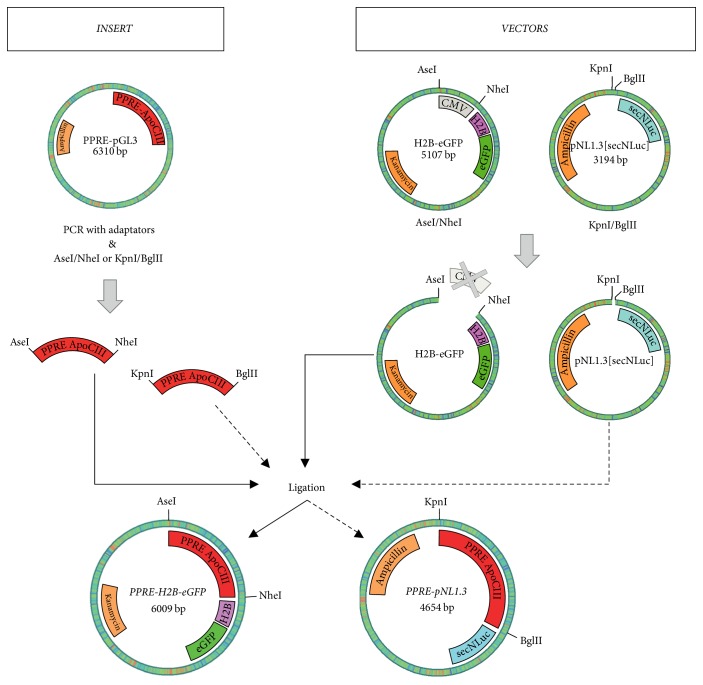
Schematic diagram of PPRE-H2B-eGFP and PPRE-pNL1.3 plasmid constructions.

The PPRE-H2B-eGFP and PPRE-pNL1.3 were constructed by a PCR-based method using appropriate primers (Table 1). The peroxisome proliferator response element (PPRE) sequence was amplified by PCR using the PPRE(ApoCIII)-pGL3 construct as a template. The amplified fragments were subcloned into the KpnI/BglII sites of the secreted NanoLuc® luciferase reporter vector, pNL1.3[secNluc] (Promega Corporation) and of AseI/NheI sites of the H2B-eGFP plasmid (Addgene #11680 [27]). The general structure of the plasmids is shown in Figure 1. The integrity of reporter plasmid sequences was confirmed by DNA sequencing. Plasmid DNA was prepared for transfection using the Qiagen Plasmid Mini and Midi Kits.

### 2.4. Treatments and PPRE-H2B-eGFP and PPRE-pNL1.3[secNluc] Transfections

The cells were treated with 1 *μ*M GW1929 (agonist, #ab142213, Abcam) or 1 *μ*M GW9662 (antagonist, #ab141125, Abcam) diluted in a culture medium containing 1% glutamine and 10% FCS (without PS) for 2 h. Before transfections, cells were washed and then incubated with Opti-MEM I medium without serum (Gibco; 200 *μ*l/12-well slide or 500 *μ*l/24-well plate). For transient transfection with PPRE-H2B-eGFP, VCT were transfected in 12-well cell culture-treated plastic slides (Ibidi) using Lipofectamine 3000 (Invitrogen) following the manufacturer's protocol. Briefly, plasmid DNA and Lipofectamine were separately diluted in Opti-MEM I medium without serum (Gibco). Then, DNA was combined with the Lipofectamine mixture and incubated for 5 minutes at room temperature. Finally, the DNA-Lipofectamine complexes were added dropwise to each well (25 *μ*l/well). For transient transfection with PPRE-pNL1.3 or pNL1.3 basic secreted luciferase reporter as control, VCT were transfected in 24-well plates (Corning) using Lipofectamine 3000 (Invitrogen) following the manufacturer's protocol. Briefly, plasmid DNA and Lipofectamine were separately diluted in Opti-MEM I medium without serum (Gibco). Then, DNA was combined with the Lipofectamine mixture and incubated for 5 minutes at room temperature. Finally the DNA-Lipofectamine complexes were added dropwise to each well (50 *μ*l/well). Four hours later, the transfected cells were washed and treated again with 1 *μ*M GW1929 or 1 *μ*M GW9662 for 48 h.

### 2.5. Immunofluorescence and Image Analysis

After 72 h of culture, cells were fixed in 4% PFA for 20 minutes at room temperature, washed in PBS, and permeabilized in 0.5% Triton X-100 in PBS for 30 minutes. Then, cells were blocked in 5% BSA IgG free and 0.1% Tween-20 in PBS for 1 h at room temperature and (i) for PPAR*γ* expression, cells were incubated with primary PPAR*γ* antibody (E-8 from Santa Cruz, 1-100) in blocking solution; (ii) for PPRE-H2B-eGFP transfected cells, only Alexa Fluor® 555 Phalloidin and DAPI labeling were performed. The next day, cells were rinsed three times with 0.1% Tween-20 in PBS (PBST), then the staining was revealed with VectaFluor™ Excel R.T.U. Antibody Kit, DyLight® 488, Anti-Mouse IgG (DK-2488, Vector Laboratories) according to the manufacturer's instructions. After three washes with PBST, cells were incubated with Alexa Fluor 555 Phalloidin (Molecular Probes, 1/200 in PBST) for 1 h, in the dark at room temperature, then washed three times in PBST, and counterstained with DAPI for 10 mins at room temperature. Finally slides were mounted with Fluoromount-G (Molecular Probes) and stored at 4°C. Confocal microscopy images (obtained with a Leica spinning-disk microscope equipped with a Plan Apo 63X/1.4 oil objective and a CoolSnap HQ2 CCD camera) were processed with ImageJ (National Institutes of Health, https://imagej.nih.gov/ij/) or Icy (Institut Pasteur, http://icy.bioimageanalysis.org/).

### 2.6. Western Blotting

After 72 h of culture, total cell extracts were prepared using NP40 Cell Lysis Buffer (Invitrogen). Protein samples were resolved by SDS-PAGE and immunoblotted with antibodies to GFP (1 *μ*g/ml, Sigma) and actin (0.2 *μ*g/ml, Sigma-Aldrich). After incubation with appropriate Alexa Fluor-conjugated secondary antibody (680 or 800 conjugate, Molecular Probes) blots were revealed by using Odyssey infrared fluorescent system (Li-Cor).

### 2.7. Nanoluciferase Assay

VCT transfected with PPRE-pNL1.3 or basic pNL1.3 were cultured in a 24-well plate for 72 hours at 37°C under the following 48 h treatments: 1 *μ*M GW1929 or 1 *μ*M GW9662. After 24 hours of treatment, 50 or 100 *μ*l of each cell supernatant was dispensed into the wells of a 96-well plate (#3610, Corning) and then frozen at −20°C. After 48 hours of treatment, the 96-well plate was thawed at room temperature and 50 or 100 *μ*l of each cell supernatant was dispensed into the wells of the same 96-well plate. The amount of secreted NanoLuc luciferase activity was determined using the Nano-Glo® Luciferase Assay (Promega Corporation) based on manufacturer's instructions. Luminescence in each well was then measured by using an EnSpire Multimode plate reader (Perkin Elmer).

### 2.8. Statistics

Three independent experiments were performed for each assay (*n* = 3). For PPRE-H2B-eGFP experiments, a minimum of 100 nuclei were analyzed per condition. For quantification, fluorescence signals were integrated over the entire nucleus. For PPRE-pNL1.3[secNluc] experiments, each condition was run in triplicate. Each luminescence reading was normalized to the corresponding nontransfected cell control. The data are expressed as the mean ± SD of the indicated number. Statistical analysis (paired *t*-test) was performed using the GraphPad Prism 6 software. Results were considered significant if *p* value < 0.05.

## 3. Results

### 3.1. PPAR*γ* Is Expressed in Villous Cytotrophoblasts (VCT) and Highly Expressed in Syncytiotrophoblasts (ST) and Plays a Role in Trophoblast Differentiation

Immunostaining, with PPAR*γ* antibody in primary trophoblast cells, showed that PPAR*γ* is expressed in VCT and ST, with a higher expression in ST (see Supplementary Figure  S1(A) in the Supplementary Material available online at https://doi.org/10.1155/2017/6139107). Furthermore, treatment with 1 *μ*M GW1929 (PPAR*γ* agonist) significantly increased fusion index whereas VCT treated with 1 *μ*M GW9662 (PPAR*γ* antagonist) significantly decreased cell fusion. This indicates that an increase in PPAR*γ* activity leads to an increase in ST formation, whereas a decrease in PPAR*γ* activity leads to a decrease in ST formation (Supplementary Figure S1(B)). Not only this but also treatment with 1 *μ*M GW1929 significantly increased hCG secretion whereas VCT treated with 1 *μ*M GW9662 significantly decreased hCG secretion. This shows that an increase in PPAR*γ* activity leads to an increase in hCG secretion, whereas a decrease in PPAR*γ* activity leads to a decrease in hCG secretion (Supplementary Figure S1(C)). In total, an increase of PPAR*γ* activity in VCT will increase cell fusion leading to more formation of multinuclear ST and so higher levels of secreted hCG.

### 3.2. PPRE-H2B-eGFP Transfection of VCT: A New Model to Visualize the Activity of PPAR*γ*

**Figure 2:**
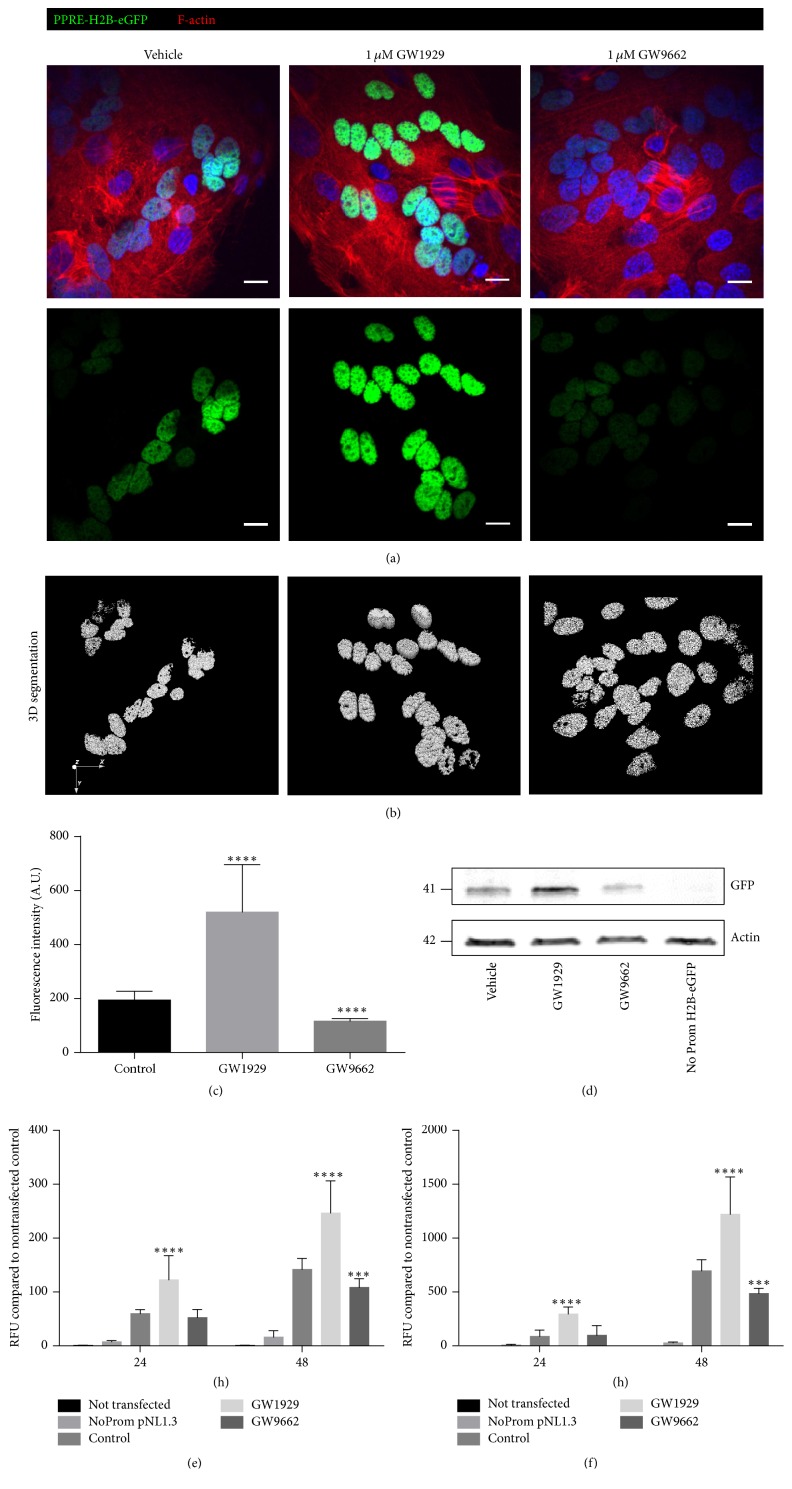
PPRE-H2B-eGFP and PPRE-pNL1.3[secNluc] transfections of VCT: new models to visualize and quantify the activity of PPAR*γ*. (a) Merged staining of PPRE-H2B-eGFP (green), cell shape using F-actin (red), and nuclei using DAPI (blue) in 48 h treated VCT with 1 *μ*M GW1929 (agonist of PPAR*γ*) or 1 *μ*M GW9662 (PPAR*γ* antagonist) compared to control (vehicle). 3D nuclei segmentation (b) and quantification (c) of PPRE-H2B-eGFP fluorescence intensity using Icy software. (d) PPRE-H2B-eGFP protein levels were assessed using western blotting. (e-f) Spectrophotometry reading of luciferase expression for 50 *μ*l (e) or 100 *μ*l (f) of supernatant from 24- and 48-hour PPRE-pNL1.3 transfected and 1 *μ*M GW1929 or 1 *μ*M GW9662 treated VCT compared to nontransfected cells (control). Values are represented as mean ± SD; ^*∗∗∗*^*p* < 0.001, ^*∗∗∗∗*^*p* < 0.0001 versus vehicle control (*n* = 3).

The transfection of primary trophoblasts with the newly constructed plasmid PPRE-H2B-eGFP showed a normal activity (normal intensity) of PPAR*γ* in the nuclei of untreated cells (Figure 2). This activity increased in the cells treated with 1 *μ*M GW1929 (PPAR*γ* agonist), whereas it decreased in cells treated with 1 *μ*M GW9662 (PPAR*γ* antagonist; Figure 2(a)). The 3D segmentation was used to quantify the signal while defining the nuclei boundaries (Figure 2(b)). The graphical representation showed a significant increase of the fluorescence intensity (by about 2.5-fold) in cells treated with 1 *μ*M GW1929, while there is a significant decrease (by about 2-fold) in cells treated with 1 *μ*M GW9662 (Figure 2(c)). Confirmed by western blotting (Figure 2(d)), this illustrates that the plasmid PPRE-H2B-eGFP is of great help to study the activity of PPAR*γ* in primary trophoblasts through visualizing methods.

### 3.3. PPRE-pNL1.3[secNluc] Transfection of VCT: A New Model to Study the Activity of PPAR*γ* without Cell Lysis

The other method that we used to study the activity of PPAR*γ* in primary trophoblasts was to transfect VCT with the new PPRE-pNL1.3 plasmid, so that the expression of PPAR*γ* leads to the secretion into the supernatant of transfected cells of the luciferase protein. Spectrophotometry reading for 50 *μ*l of supernatant from 24 and 48 hours of PPRE-pNL1.3 transfected/GW treated VCT showed an increase in luminescence intensity with 1 *μ*M GW1929 (PPAR*γ* agonist) and a decrease in luminescence intensity with 1 *μ*M GW9662 (PPAR*γ* antagonist) compared to nontransfected cells with more relevant results in cells that were treated for 48 hours (Figure 2(e)). On the other hand, spectrophotometry reading for 100 *μ*l of supernatant from 24- and 48-hour PPRE-pNL1.3 transfected/GW treated VCT showed an increase in luminescence intensity with 1 *μ*M GW1929 (PPAR*γ* agonist) and a decrease in luminescence intensity with 1 *μ*M GW9662 (PPAR*γ* antagonist) compared to nontransfected cells with more relevant results in cells treated for 48 hours (Figure 2(f)). Altogether these results showed that the PPRE-pNL1.3 is another great tool to study PPAR*γ* activity in primary trophoblasts without cells lysis, so that they can be further used for other experiments.

### 3.4. PPRE-H2B-eGFP Model Works in Different Types of Cells

To confirm that PPRE-H2B-eGFP worked on other cell types, an immunostaining with a PPAR*γ* antibody was performed after transfection on human or bovine cells available in the laboratory, namely, human trophoblast primary cells, human BEWO trophoblast cell line, human placental mesenchymal cells, or bovine extraembryonic mesoderm cells (bXMCs), was done. Immunocytofluorescence data evidenced that the four cell types expressed PPAR*γ* and that PPAR*γ* transcriptional activity was detectable in their nuclei due to the fluorescence of translated eGFP reporter gene (Supplementary Figure S2).

## 4. Discussion

In brief, the current work demonstrated the validity of two novel PPRE reporter systems for the in vitro study of PPAR*γ* activity. On the one hand, our novel assay using a secreted nanoluciferase is highly sensitive requiring only a small amount of culture medium to evaluate changes in luciferase activity over time. On the other hand, our GFP reporter can be used to monitor PPAR*γ* activity through live imaging. Moreover, these tools can easily be adapted to high-throughput screening of compounds (96-well or 384-well plates) which could identify agonist or antagonist effects on PPAR*γ* activity on the cells of interest.

## Supplementary Material

Figure S1: PPARγ: expression and role during trophoblast differentiation (fusion, hCG secretion). A) PPARγ in villous cytotrophoblast (VCT) and syncytiotrophoblast (ST). To the left- merged immunostaining in VCT and ST for PPARγ expression using PPARγ antibody (green), cell shape using F-actin (red) and nuclei using DAPI (blue). To the right- Immunostaining showing the differential expression of PPARγ between VCT and ST. PPARγ is highly expressed in ST. B) Fusion index of 48h-treated VCT with 1µM GW1929 (agonist of PPARγ) or 1µM GW9662 (PPARγ antagonist) compared to control (vehicle). C) hCG secretion of 48h-treated VCT with 1µM GW1929 (agonist of PPARγ) or 1µM GW9662 (PPARγ antagonist) compared to control (vehicle). Values are represented as mean ±S.D; ∗∗∗P < 0.001, ∗∗∗∗P < 0.0001 versus vehicle control (n=5).
Figure S2: PPRE-H2B-eGFP works in different cell types and species. Left panels- PPARγ is expressed in human primary cells (VCT/ST), BEWO, and mesenchyme, as well as in bovine extra-embryonic mesoderm cells. Displayed are the merged immunostaining for PPARγ expression using PPARγ antibody (green), cell shape using F-actin (red) and nuclei using DAPI (blue). Middle panels- only PPARγ expression (green). Left panels- PPARγ activity, PPRE-H2B-eGFP (green) and nuclei (Hoechst, blue).

## Figures and Tables

**Table 1:** Primers used to amplify DNA fragments by PCR for vector construction, confirmation, and generation of cloned fragments.

Primer name	Sequence
PPRE-*Ase*I	ATTAATAATGCCTGCAGGTCAATTCTG
PPRE-*Nhe*I	GCTAGCGATCGCAGATCCTCTAGAGTCC
PPRE-*Kpn*I	CGGGGTACCATGCCTGCAGGTCAATTCTG
PPRE-*Bgl*II	GAAGATCTGATCGCAGATCCTCTAGAGTCC
pNL_F	AGGCTGTCCCCAGTGCAAGT
pNL_R	CGGATTGCCAAGCTTGGC
H2B_F	CACCATGCCAGAGCCAG
H2B_R	CTTAGCGCTGGTGTACTTG
GFP_F	CATGGTCCTGCTGGAGTTCGTG
GFP_R	CGTCGCCGTCCAGCTCGACCAG

Restriction enzyme sites added to primer 5′-ends are underlined.

## Data Access

The PPRE-H2B-eGFP and PPRE-pNL1.3[secNluc] plasmids described in this report have been submitted for inclusion in the nonprofit plasmid repository Addgene (https://www.addgene.org/) and will be available as Plasmid #84393 and Plasmid #84394, respectively.
