# Supplementary material for: New Transcriptional Reporters to Quantify and Monitor PPARγ Activity
Source: PPAR Res. 2017 Nov 1;2017:6139107. doi: 10.1155/2017/6139107 (PMC5684601; doi:10.1155/2017/6139107)
Supplement: Supplementary file 1 — Figure S1: PPARγ: expression and role during trophoblast differentiation (fusion, hCG secretion). A) PPARγ in villous cytotrophoblast (VCT) and syncytiotrophoblast (ST). To the left- merged immunostaining in VCT and ST for PPARγ expression using PPARγ antibody (green), cell shape using F-actin (red) and nuclei using DAPI (blue). To the right- Immunostaining showing the differential expression of PPARγ between VCT and ST. PPARγ is highly expressed in ST. B) Fusion index of 48h-treated VCT with 1µM GW1929 (agonist of PPARγ) or 1µM GW9662 (PPARγ antagonist) compared to control (vehicle). C) hCG secretion of 48h-treated VCT with 1µM GW1929 (agonist of PPARγ) or 1µM GW9662 (PPARγ antagonist) compared to control (vehicle). Values are represented as mean ±S.D; ∗∗∗P < 0.001, ∗∗∗∗P < 0.0001 versus vehicle control (n=5). Figure S2: PPRE-H2B-eGFP works in different cell types and species. Left panels- PPARγ is expressed in human primary cells (VCT/ST), BEWO, and mesenchyme, as well as in bovine extra-embryonic mesoderm cells. Displayed are the merged immunostaining for PPARγ expression using PPARγ antibody (green), cell shape using F-actin (red) and nuclei using DAPI (blue). Middle panels- only PPARγ expression (green). Left panels- PPARγ activity, PPRE-H2B-eGFP (green) and nuclei (Hoechst, blue). [file 6139107.f1.pdf]

Figure S1

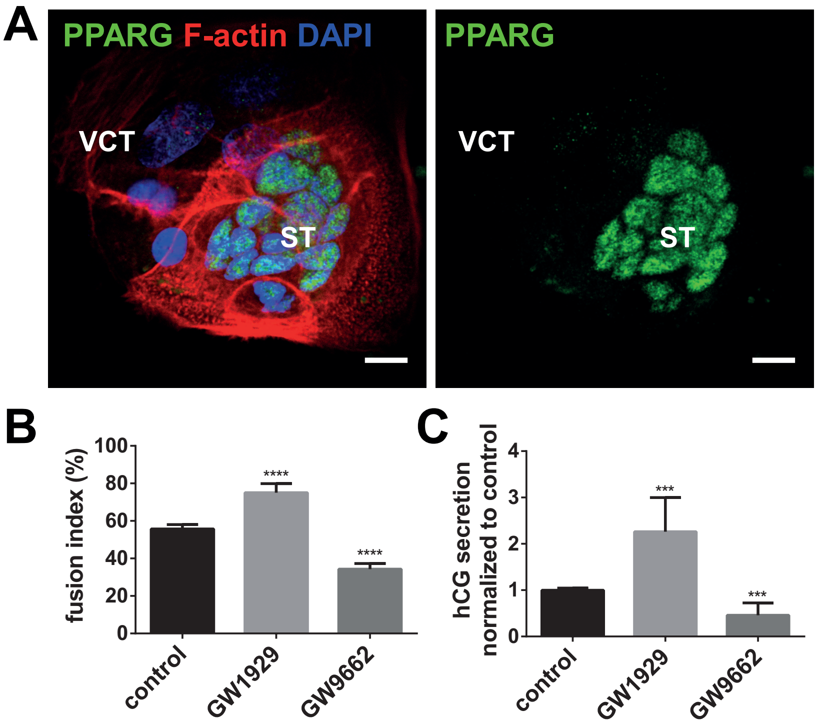

Figure S1. PPARγ: expression and role during trophoblast differentiation (fusion, hCG secretion). A) PPARγ in villous cytotrophoblast (VCT) and syncytiotrophoblast (ST). To the left- merged immunostaining in VCT and ST for PPARγ expression using PPARγ antibody (green), cell shape using F-actin (red) and nuclei using DAPI (blue). To the right- Immunostaining showing the differential expression of PPARγ between VCT and ST. PPARγ is highly expressed in ST. B) Fusion index of 48h-treated VCT with 1μM GW1929 (agonist of PPARγ) or 1μM GW9662 (PPARγ antagonist) compared to control (vehicle) C) hCG secretion of 48h-treated VCT with 1μM GW1929 (agonist of PPARγ) or 1μM GW9662 (PPARγ antagonist) compared to control (vehicle). Values are represented as mean ±S.D; *** $P < 0.001$ versus vehicle control; **** $P < 0.0001$ (n=5).

Figure S2

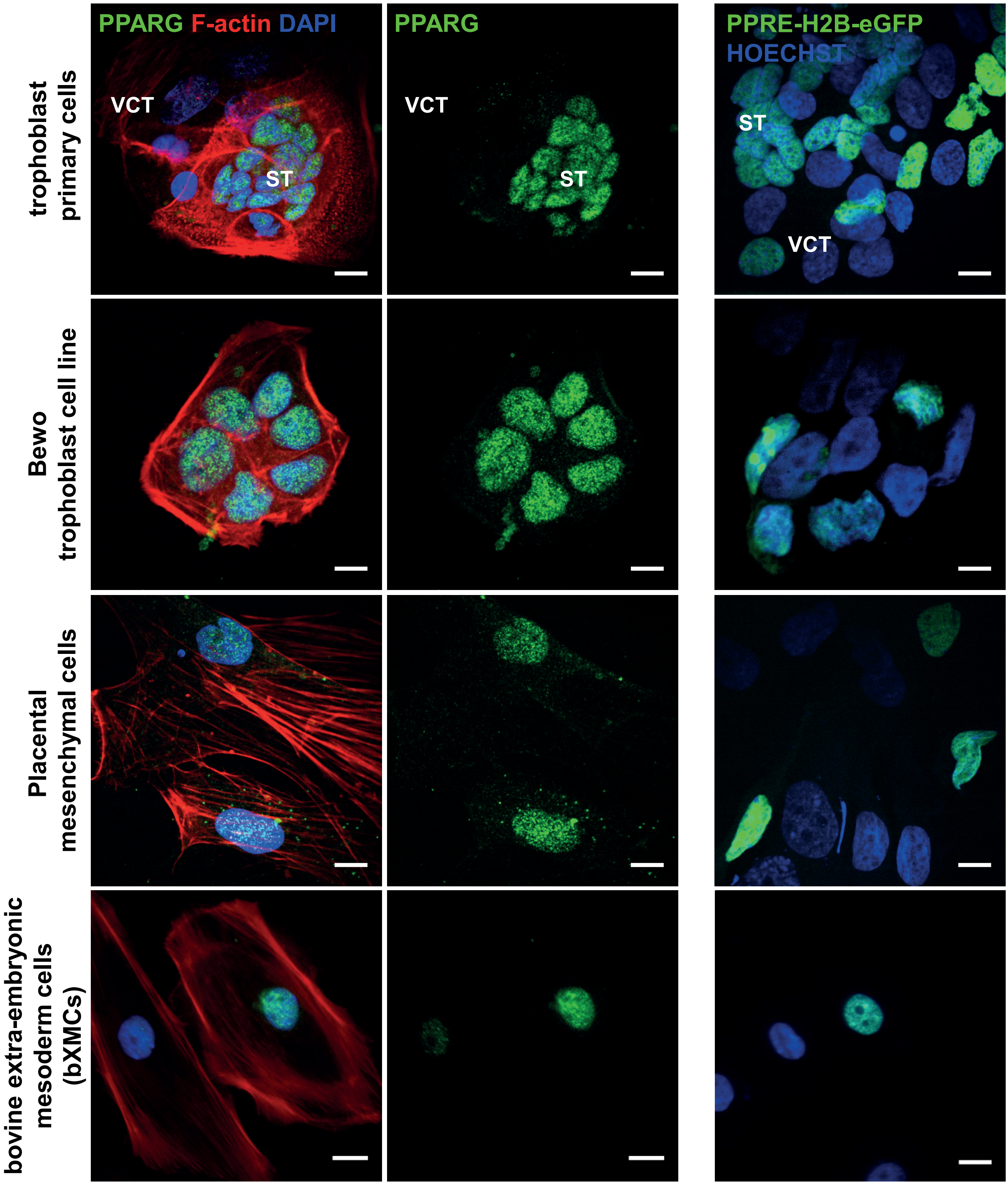
PPARG F-actin DAPI
PPARG
PPRE-H2B-eGFP
HOECHST
trophoblast primary cells
VCT
ST
VCT
ST
ST
VCT
Bewo trophoblast cell line
Placental mesenchymal cells
bovine extra-embryonic mesoderm cells (bXMCs)

Figure S2. PPRE-H2B-eGFP works in different types of cells and different species. Left panels- PPARγ is expressed in human primary cells (VCT/ST), BEWO, and mesenchyme, as well as in bovine extra-embryonic mesoderm cells. Displayed are the merged immunostaining for PPARγ expression using PPARγ antibody (green), cell shape using F-actin (red) and nuclei using DAPI (blue). Middle panels- only PPARγ expression (green). Left panels- PPARγ activity, PPRE-H2B-eGFP (green) and nuclei (Hoechst, blue).
